# Meta‐analysis of continuous outcomes: Using pseudo IPD created from aggregate data to adjust for baseline imbalance and assess treatment‐by‐baseline modification

**DOI:** 10.1002/jrsm.1434

**Published:** 2020-07-25

**Authors:** Katerina Papadimitropoulou, Theo Stijnen, Richard D. Riley, Olaf M. Dekkers, Saskia le Cessie

**Affiliations:** ^1^ Clinical Epidemiology Leiden University Medical Center Leiden The Netherlands; ^2^ Data Science and Biometrics Danone Nutricia Research Utrecht The Netherlands; ^3^ Biomedical Data Sciences Leiden University Medical Center Leiden The Netherlands; ^4^ Centre for Prognosis Research, Research Institute for Primary Care & Health Sciences Keele University Keele UK

**Keywords:** ANCOVA, meta‐analysis, pseudo individual participant data, sufficient statistics

## Abstract

Meta‐analysis of individual participant data (IPD) is considered the “gold‐standard” for synthesizing clinical study evidence. However, gaining access to IPD can be a laborious task (if possible at all) and in practice only summary (aggregate) data are commonly available. In this work we focus on meta‐analytic approaches of comparative studies where aggregate data are available for continuous outcomes measured at baseline (pre‐treatment) and follow‐up (post‐treatment). We propose a method for constructing pseudo individual baselines and outcomes based on the aggregate data. These pseudo IPD can be subsequently analysed using standard analysis of covariance (ANCOVA) methods. Pseudo IPD for continuous outcomes reported at two timepoints can be generated using the sufficient statistics of an ANCOVA model, i.e., the mean and standard deviation at baseline and follow‐up per group, together with the correlation of the baseline and follow‐up measurements. Applying the ANCOVA approach, which crucially adjusts for baseline imbalances and accounts for the correlation between baseline and change scores, to the pseudo IPD, results in identical estimates to the ones obtained by an ANCOVA on the true IPD. In addition, an interaction term between baseline and treatment effect can be added. There are several modeling options available under this approach, which makes it very flexible. Methods are exemplified using reported data of a previously published IPD meta‐analysis of 10 trials investigating the effect of antihypertensive treatments on systolic blood pressure, leading to identical results compared with the true IPD analysis and of a meta‐analysis of fewer trials, where baseline imbalance occurred.

**Abbreviations**
AD: aggregate data
CI: confidence interval
IPD: individual patient (participant) data
RCT: randomized controlled trial

**Highlights**

1 **What is already known?**

The meta‐analysis of IPD has been advocated as the “gold‐standard” of evidence synthesis for many years. The generally preferred method to analyse IPD with continuous measurements at baseline and follow‐up is the linear mixed effects ANCOVA model. However access to IPD is often impossible. Researchers thus resort in an AD meta‐analysis where in case of baseline imbalances, the treatment effects, derived by other methods than ANCOVA, may be biased.

2 **What is new?**

We provide an algorithm which makes use of summary reported AD of continuous measurements at baseline and follow up for to construct pseudo IPD. These pseudo IPD can be analysed in the same way as the original IPD using ANCOVA, producing identical results. Therefore we can adjust for baseline imbalances between treatment and control groups and explore interactions between baseline values and treatment effects. The results of the analysis under our proposed algorithm in the example dataset where the true IPD have been synthesized, were found to be identical to the analysis of the original true IPD.

3 **What is the potential impact for RSM readers outside the author's field?**

To enable reproducibility and dissemination of the method, we have provided implementation code of the algorithm both in R and SAS. Meta‐analysis is a statistical technique undertaken by researchers from various fields and thus being able to use the provided code in easily accessible free and commercial software can only improve the quality of their work.

## 1 INTRODUCTION

Meta‐analysis methods of individual participant data or individual patient data (IPD) are considered the “gold‐standard” for clinical studies' evidence synthesis.^1^, ^2^, ^3^, ^4^ IPD meta‐analysis has several advantages over the traditional aggregate data (AD) meta‐analysis approach, which synthesizes summary statistics per study, often retrieved from published sources. For example when continuous outcomes are available at baseline and follow‐up, IPD meta‐analysis enables the meta‐analyst to perform adjustments for baseline imbalances and detailed explorations of treatment‐covariate interactions.^5^, ^6^, ^7^ In addition, it comes with a large toolbox of methods and greater flexibility to analyze the data in an one‐stage or a two‐stage approach.^8^, ^9^, ^10^, ^11^

There are, however, challenges as access to IPD can be problematic because of time and cost constraints and privacy issues, and often it is not feasible to retrieve the IPD of all studies to be synthesized. It is possible to generate/back‐calculate IPD for different types of AD, such as for binary, ordinal and time to event outcomes.^12^, ^13^, ^14^, ^15^ For aggregate data of continuous outcomes reconstructing the original outcome values is not possible. However, we recently proposed an algorithm to construct pseudo IPD for an one‐stage meta‐analysis with one continuous outcome, using the sufficient statistics for linear mixed models, i.e.,, group means, standard deviations and sample sizes.^16^ In this way the analysis using the pseudo IPD yields exactly the same results as the analysis of the original IPD. The pseudo IPD approach allowed more flexible modeling, using standard linear mixed model software, for example enabling common or different residual variances for treatment and control groups in each study.

In this paper we extend the original method of creating pseudo IPD from reported AD to the situation where continuous outcomes are reported both at baseline and follow‐up. We discuss how pseudo IPD can be derived, taking the correlation between baseline and follow‐up/final measurements into account, using the summary observed group means, standard deviations at baseline and post‐treatment, and the group correlation of the baseline and post‐treatment values (or equivalently the standard deviations of the difference between baseline and post‐baseline values in both groups). These summary measures are the *sufficient statistics* for an analysis of covariance (ANCOVA) approach under the linear mixed model (LMM) framework. The generated pseudo IPD can be analysed using standard software for linear mixed models, and a linear mixed model analysis of the pseudo IPD will yield identical results to the ones obtained when it is applied on the original IPD.

We describe the advantages of this approach, compared with the standard methods to synthesize aggregate baseline and follow‐up data: using mean *follow‐up* (post‐treatment/final) scores, ignoring the baseline values and mean *change scores*, subtracting the follow‐up value from the baseline.^17^, ^18^

It is possible to perform a meta‐analysis in an one‐stage or a two‐stage approach using the pseudo IPD, using the toolbox of available IPD methods.^8^, ^9^, ^10^, ^11^ A plethora of modeling options is available and we discuss several options, assuming stratified and random study intercepts and random treatment effect models.

The flexibility of the linear mixed modeling framework makes it possible to correct for potential baseline imbalances. Although imbalance at baseline is not expected in a randomized trial, it can occur by chance, particularly in small trials^19^ or due to flaws in the randomization process.^20^

Treatment effects may also differ between patients, depending on their baseline values. For example, in a trial for hypertension, patients with low systolic blood pressure at baseline are expected to experience less improvement after administration of treatment, compared with patients having high baseline pressure values. Similarly, severely depressed patients with high values on a depression score may profit more from treatment than patients with mild depression. When generating and analyzing pseudo IPD using an ANCOVA approach we can cope with the correlation between the baseline value and the change score by introducing an interaction term between the baseline measurement and the treatment effect. In this way treatment heterogeneity depending on the baseline values can be further explored.

The paper is organized as follows. In Section 2 we introduce two illustrating meta‐analysis datasets: one in hypertension where group‐level AD of systolic blood pressure (SBP) at baseline and at follow‐up for anti‐hypertensive treatments vs placebo/no treatment are available from a previous IPD meta‐analysis publication^21^ and a second example where active vs sham treatments in obstructive sleep apnea are compared and baseline imbalance occurred between the treatment groups.^22^ In Sections 3 and 4, we describe some of the existing modeling options for one‐stage and two‐stage IPD meta‐analyses, respectively, including models for treatment‐by‐baseline interaction. In Section 5, we explain how pseudo IPD baselines and outcomes can be generated from the aggregate continuous data in the case of correlated baseline and final measurements. In Section 6, we apply our proposed method to the hypertension dataset in/excluding an investigation of the interaction between baseline and treatment and compare the results with those obtained when using the original IPD as previously reported in the work of Riley et al^21^, ^23^ and with standard two‐stage methods on the AD. In addition, we apply the pseudo IPD approach on the sleep apnea dataset and compare the results of the pseudo IPD ANCOVA models, while varying group‐correlations coefficients (as sensitivity analysis), with change scores AD meta‐analysis. Brief final comments are provided in Section 7.

## 2 ILLUSTRATING EXAMPLES

### 2.1 Aggregate data from 10 trial in hypertension with baseline imbalance and artificial baseline imbalance

We use the reported aggregate data for studies originally contained in an IPD meta‐analysis of Wang et al,^24^ and subsequently analysed by Riley et al^21^ investigating the effect of hypertension treatments on systolic blood pressure (SBP). The authors included IPD of trials comparing antihypertensive treatments against placebo/no treatment.^25^, ^26^, ^27^, ^28^ A total of 28 851 patients from 10 trials were included. Each trial measured blood pressure at baseline and after treatment. The aggregate data for each trial, including the mean, standard deviation and correlation of the baseline and the final SBP values (in mmHg) are shown in Table 1. Riley et al^21^ compared several IPD and AD meta‐analytic approaches to estimate the summary treatment effect of antihypertension treatments in reducing SBP. In this article, we re‐analyze these data using only the aggregate group means, standard deviations and correlations of the baseline and the final values and apply our algorithm to generate pseudo IPD. We also perform standard AD meta‐analysis using change scores and provide a comparison of the different methods. Riley et al^21^ explored the effect of large baseline imbalance by modifying the original hypertension dataset. This was achieved by subtracting 5 mmHg from the baseline and final SBP values of patients in the treatment group of trials 1 and 2; 20 mmHg of patients in the treatment group of trials 4 and 5 and 10 mmHg of the baseline and final values of patients in the treatment group of trial 6 accordingly, such that five studies have lower baseline values in the treatment group compared with the control group. We also demonstrate our method on the aggregate version of this modified dataset.

**TABLE 1:** Aggregate data of the 10 hypertension trials included in the meta‐analysis of Wang et al^24^ as reported by Riley et al^21^

				SBP final (mmHg)		SBP final (mmHg)			
		Number of subjects		Treatment	Control	Treatment	Control	Correlation (SBP baseline, SPB final)	
ID	Trial name	Treatment	Control	Mean (SD)	Mean (SD)	Mean (SD)	Mean (SD)	Treatment	Control
1	ATMH	780	750	152.28 (15.25)	153.05 (15.73)	132.85 (16.72)	139.75 (17.85)	0.265	0.284
2	HEP	150	199	189.94 (16.15)	191.55 (17.64)	165.06 (20.03)	179.89 (22.15)	0.335	0.331
3	EWPHE	90	82	177.33 (15.85)	178.23 (15.06)	156.88 (21.26)	170.45 (26.91)	0.462	0.534
4	HDFP	2427	2370	151.68 (19.83)	151.00 (19.53)	130.09 (19.25)	138.54 (21.26)	0.337	0.408
5	MRC‐1	3546	3445	156.60 (16.09)	156.65 (15.96)	135.49 (16.32)	144.25 (17.58)	0.346	0.416
6	MRC‐2	1314	1337	182.19 (12.63)	182.13 (12.73)	153.99 (20.13)	164.58 (19.71)	0.178	0.137
7	SHEP	2365	2371	170.49 (9.5)	170.12 (9.24)	145.10 (19.05)	156.24 (20.12)	0.315	0.253
8	STOP	137	131	194.68 (12.21)	194.15 (11.16)	171.46 (19.29)	189.11 (21.9)	0.177	0.414
9	Sy‐Chi	1252	1139	170.73 (10.9)	170.25 (11.41)	150.2 (15.84)	156.55 (16.86)	0.199	0.347
10	Sy‐Eur	2398	2297	173.75 (9.86)	173.94 (10.07)	154.87 (16.31)	165.24 (16.33)	0.319	0.431

Abbreviations: ATMH, Australian Trial in Mild Hypertension; HDFP, Hypertension Detection and Follow‐up Programme; EWPHE, European Working Party on High Blood Pressure in the Elderly; MRC, Medical Research Council; SBP, systolic blood pressure; SD, standard deviation; SHEP, Systolic Hypertension in the Elderly Programme; STOP, Swedish Trial in Old Patients with Hypertension; Sy‐Chi, Systolic Hypertension in China; Sy‐Eur, Systolic Hypertension in Europe.

### 2.2 Aggregate data from eight trials in obstructive sleep apnea with baseline imbalance

**FIGURE 1:**
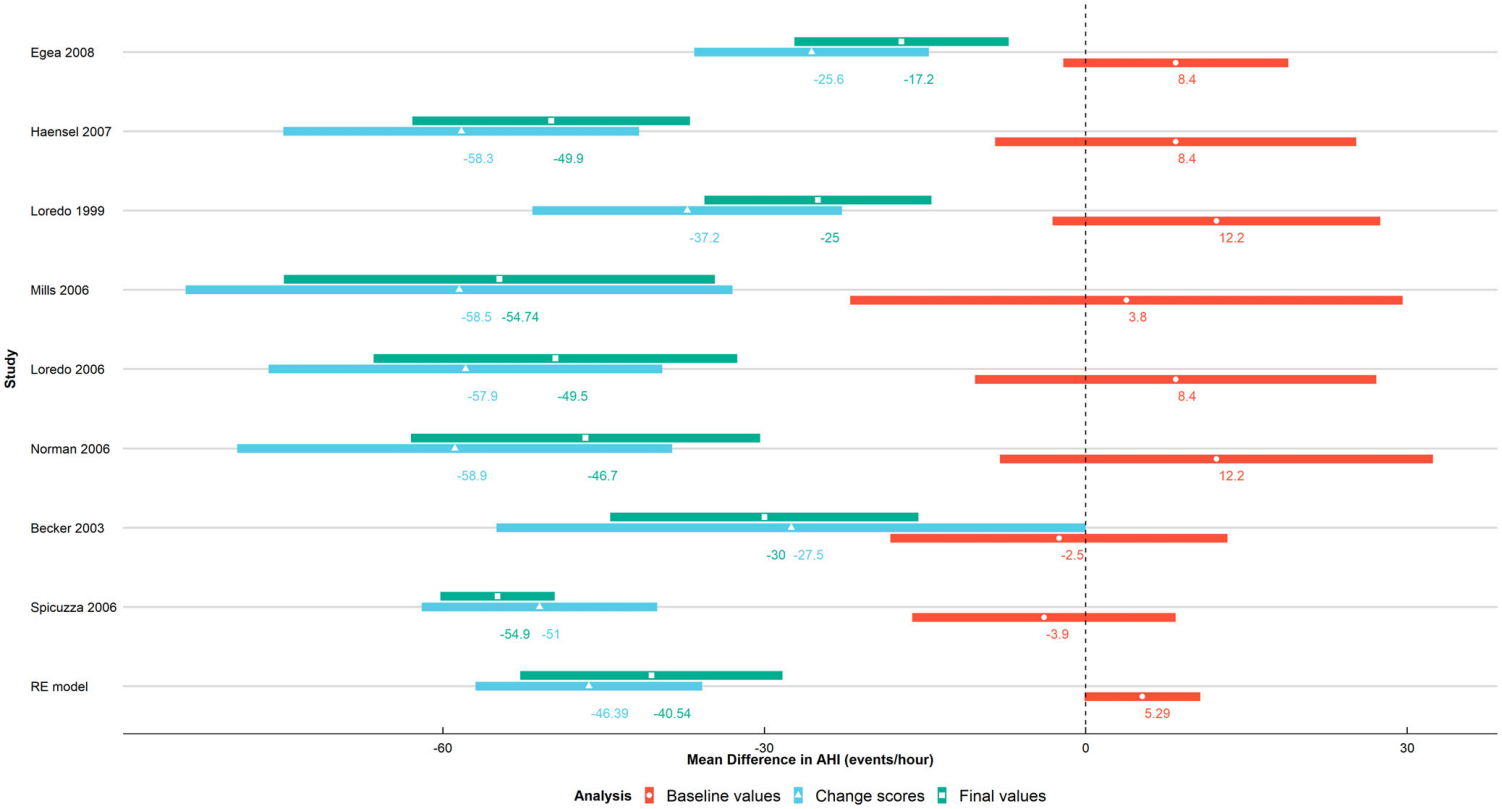
Obstructive sleep apnea meta‐analysis example: forest plot of three different summary measures: A, difference in final values between mean AHI in the active CPAP group and mean AHI in the sham CPAP group (green/square); B, between groups difference in mean change from baseline (blue/triangle); C, between groups difference in mean AHI score at baseline (red/circle). The estimates at the bottom of the plot correspond to the standard random effects meta‐analysis results [Colour figure can be viewed at wileyonlinelibrary.com]

Aggregate data from a review of treatments for obstructive sleep apnea in adults^22^ were used. We focus on a meta‐analysis summarizing the treatment effect of an active continuous positive airway pressure (CPAP) device vs a sham CPAP. Eight studies, of in total 311 patients, recorded the apnea‐hypopnea index (AHI), which is defined as the number of apnea and hypoapnea events divided by the total hours of sleep, at baseline and follow‐up. The authors^22^ estimated a statistically significant mean difference in change scores of AHI between active CPAP and sham, favoring CPAP (difference −46 events/hour 95% CI: [−57, −36]; blue/triangle, Figure 1). We re‐analysed these data, taking into account the considerable baseline imbalance which occurred between the treatment groups (difference of 5 events/hour, 95% CI [0, 11]—the subjects randomized in the active CPAP arm suffered more severely from sleep apnea; red/circle, Figure 1), and explored whether patients with higher AHI at baseline benefitted more from treatment. For comparison purposes, we have additionally included the summary estimates of the final values analysis, which is not preferred due to baseline imbalance (green/square, Figure 1).

## 3 ONE‐STAGE IPD META‐ANALYSIS USING LMM

In this section we introduce notation and modeling options for an one‐stage meta‐analysis of IPD of studies measuring continuous outcomes as baseline and follow‐up. The data we consider have the following format: let *Y*_*Bij*_ denote the *continuous outcome of interest* (ie, SBP) at baseline/pre‐treatment of patient *j* in study *i*(1, …, *N*) and *Y*_*Fij*_ the outcome, of each patient post‐treatment (at follow‐up). Also, let *X*_*ij*_ be a dummy variable to indicate the treatment group; *X*_*ij*_ = 1 for patients in the treatment group and 0 for patients in the control group, respectively. There are many IPD meta‐analysis ANCOVA type model options. A number of them are presented in this section; a similar description of the ANCOVA model can be found in Burke et al.^8^

### 3.1 Analysis of covariance

#### 3.1.1 Stratified study model

An analysis of covariance (ANCOVA) model, with study‐specific stratified intercepts and stratified adjustment terms for baseline measurements may be written as follows:

(1) $$Y_{\mathrm{Fij}}=\beta_{0i}+\left(\beta_{1}+b_{1i}\right)X_{\mathrm{ij}}+\beta_{2i}\left(Y_{\mathrm{Bij}}-{\bar{Y}}_{\mathrm{Bi}}\right)+{ɛ}_{\mathrm{ij}},$$

where *β*_0*i*_ is the mean outcome in the control group in study *i* for individuals with the mean baseline value, *β*_1_ the summary (average) treatment effect and *β*_2*i*_ is the study‐specific adjustment term for baseline values. A random effect *b*_1*i*_ is added to the overall treatment effect, which is assumed to be normally distributed with mean 0 and between‐study variance equal to $\tau_{1}^{2}$. Although a random treatment effect is preferred, one can assume a common (fixed) treatment effect by constraining $\tau_{1}^{2}=0$. There are several modeling options for the variance of the within‐study residuals, ɛ_*ij*_, on which we elaborate later on.

#### 3.1.2 Random study model

An alternative approach to using stratified study intercepts and slopes is to assume a random intercept and a random baseline adjustment effect, resulting in the following ANCOVA model:

(2) $$Y_{\mathrm{Fij}}=\left(\beta_{0}+b_{0i}\right)+\left(\beta_{1}+b_{1i}\right)X_{\mathrm{ij}}+\left(\beta_{2}+b_{2i}\right)\left(Y_{\mathrm{Bij}}-{\bar{Y}}_{\mathrm{Bi}}\right)+{ɛ}_{\mathrm{ij}},$$

$$\text{where}\left[\begin{array}{c} b_{0i}\\ b_{1i}\\ b_{2i} \end{array}\right]∼\mathrm{MVN}\left(\left[\begin{array}{c} 0\\ 0\\ 0 \end{array}\right],\left[\begin{array}{ccc} \tau_{0}^{2} & \tau_{01} & \tau_{02}\\ \tau_{01} & \tau_{1}^{2} & \tau_{12}\\ \tau_{02} & \tau_{12} & \tau_{2}^{2} \end{array}\right]\right)$$

Parameters are as in Equation (1), except for a random study intercept and a baseline adjustment coefficient; with $\tau_{1}^{2}$ denoting the variance of the treatment effect. In the literature, is it often assumed that the random effects are independent (ie, *τ*_*ij*_ = 0 for *i* ≠ *j*), although under the LMM it is possible to estimate their covariances.

### 3.2 ANCOVA including treatment‐by‐baseline interaction

To investigate potential treatment effect modification by the baseline value, the Equations (1) and (2) can be extended by including the interaction term between baseline and treatment effect. The stratified study model (1) incorporating the “treatment‐covariate interaction” is as follows:

(3) $$Y_{\mathrm{Fij}}=\beta_{0i}+\left(\beta_{1}+b_{1i}\right)X_{\mathrm{ij}}+\beta_{2i}\left(Y_{\mathrm{Bij}}-{\bar{Y}}_{\mathrm{Bi}}\right)+\left(\beta_{3}+b_{3i}\right)\left[\left(Y_{\mathrm{Bij}}-{\bar{Y}}_{\mathrm{Bi}}\right)X_{\mathrm{ij}}\right]+\beta_{4i}\left({\bar{Y}}_{\mathrm{Bi}}X_{\mathrm{ij}}\right)+{ɛ}_{\mathrm{ij}}$$

While the other parameters are as in Equation (1), *β*_3_ denotes the mean increase in treatment effect for a one‐unit increase in the baseline values and the random effect *b*_3*i*_ allows for between studies heterogeneity in the treatment‐covariate interaction. This estimate reflects the within‐trial interaction effect and *β*_4*i*_ estimates the increase in the treatment effect associated with a one‐unit increase between the mean baseline of two studies, which reflects the across‐trial interaction. Centering the baseline values and appropriately separating within‐ and across trial‐associations avoids ecological bias, a phenomenon where the associations are erroneously equated.^29^ Note that if the $\beta_{4i}\left({\bar{Y}}_{\mathrm{Bi}}X_{\mathrm{ij}}\right)$ is omitted from model (4), then the interaction term will reflect a weighted average of *β*_3_ and the magnitude of the ecological bias.^30^

Similarly, Equation (2) can be extended yielding a random study ANCOVA model allowing for the interaction between baseline and treatment, which is formulated as follows:

(4) $$Y_{\mathrm{Fij}}=\left(\beta_{0}+b_{0i}\right)+\left(\beta_{1}+b_{1i}\right)X_{\mathrm{ij}}+\left(\beta_{2}+b_{2i}\right)\left(Y_{\mathrm{Bij}}-{\bar{Y}}_{\mathrm{Bi}}\right)+\left(\beta_{3}+b_{3i}\right)\left[\left(Y_{\mathrm{Bij}}-{\bar{Y}}_{\mathrm{Bi}}\right)X_{\mathrm{ij}}\right]+\beta_{4i}\left({\bar{Y}}_{\mathrm{Bi}}X_{\mathrm{ij}}\right)+{ɛ}_{\mathrm{ij}}$$

This model has four random effects (*b*_0*i*_, *b*_1*i*_, *b*_2*i*_, *b*_3*i*_), the covariance matrix of which may either be completely unspecified or may be modeled, for example by assuming independence of the different random effects.

Although, many other modeling specifications are possible, in this work we consider models (1), (2), (3), (4)).

### 3.3 Within‐study residual variances

The within‐study residuals ɛ_*ij*_ are assumed to follow a normal distribution with mean 0. The within‐study residual variance $\sigma_{\mathrm{ik}}^{2}$ may depend on the study *i* and group *k*. We explore *four structures* for modeling $\sigma_{\mathrm{ik}}^{2}$: all variances assumed different (arm‐ and study‐specific): ɛ_*ik*_ $∼N\left(0,\sigma_{\mathrm{ik}}^{2}\right)$, study‐specific variances: $\sigma_{\mathrm{ik}}^{2}=\sigma_{i.}^{2}$, one variance for control and one variance for treated group $\sigma_{\mathrm{ik}}^{2}=\sigma_{.k}^{2}$, which are the same for all studies and one overall variance: $\sigma_{\mathrm{ik}}^{2}=\sigma^{2}$.

## 4 TWO‐STAGE IPD META‐ANALYSIS APPROACH

Instead of modeling all IPD in one model, in practice it may be more convenient to use a two‐step approach. In the first stage, a separate ANCOVA is fitted in each of the studies *i* = 1 to *N*.

(5) $$Y_{\mathrm{Fij}}=\beta_{0i}+\beta_{1i}X_{\mathrm{ij}}+\beta_{2i}Y_{\mathrm{Bij}}+{ɛ}_{\mathrm{ij}}$$

This yields *N* treatment effects ${\hat{\beta }}_{1i}$ with standard errors *se*_*i*_.

At the second stage a common (fixed)‐effect or random‐effects meta‐analysis is run on the estimated study‐specific *β*_1*is*_.

In principle, the one‐stage and two‐stage approaches produce very similar results yet minor differences may arise as the former estimates the within‐study residual variances simultaneously with *β*_1*i*_ and $\tau_{1}^{2}$ while under the two‐stage approach the within‐study residual variances are estimated separately as seen in Equation (5) and independently of *β*_1*i*_ and $\tau_{1}^{2}$ in the second stage. In particular, the stratified study one‐stage model (1) and two‐stage IPD meta‐analysis approaches will yield very similar results, under the same underlying (modeling) assumptions, for example, equal variance for treatment and control within studies.^8^ For small sample sizes the results may deviate slightly. Equation (5) can also be extended to estimate the interaction between baseline values and treatment effect by introducing an interaction term similar to term *β*_3_ from Equation (3).

## 5 CONSTRUCTION OF PSEUDO IPD FROM AGGREGATE DATA

In our previous work we developed a method to generate pseudo IPD for a single continuous outcome per subject without baseline values.^16^ The method generates data with the same observed means, standard deviations and sample sizes, the so‐called pseudo IPD. Because the means and standard deviations are the sufficient statistics, the likelihood function for the IPD, using the linear mixed model is identical to the likelihood of the unknown true IPD. This means that analyzing the pseudo IPD with LMM will yield identical results to the analysis of the true IPD.

In this article we extend our method to creating pseudo IPD from available aggregate data for a continuous outcome, reported at two timepoints, at baseline and follow‐up. Appropriate sufficient statistics for an analysis of covariance (ANCOVA) approach are, for each study separately, the means and standard deviations of the continuous outcome at baseline and follow‐up in each group, together with the group correlation of the baseline and follow‐up values. Our premise is to create pseudo IPD that have exactly these sample means, standard deviations, and correlations, so that the subsequent pseudo IPD meta‐analysis will produce the same results as if the original IPD were analysed.

The algorithm to construct pseudo data for each of the studies and groups, with exactly the same mean, standard deviation and group correlation between baseline and follow‐up measurement is as follows: let in a certain study arm, ${\bar{Y}}_{B}$, *sd*_*B*_ and ${\bar{Y}}_{F}$, *sd*_*F*_ be the observed means and SDs at baseline and follow‐up, respectively and let *r* be the correlation between baseline and follow‐up measurement, and let *n* be the sample size. Then for *each group in each study* separately, execute the following steps:

1. Simulate two samples $Y_{i1}^{*}\left(i=1,\ldots ,n\right)$ and $Y_{i2}^{*}\left(i=1,\ldots ,n\right)$, from a certain distribution, for example a standard normal distribution.
2. Standardize both samples to obtain ${\bar{Y}}_{1}^{*}=0$ and ${\bar{Y}}_{2}^{*}=0$, and ${\mathrm{sd}}_{1}^{*}={\mathrm{sd}}_{2}^{*}=1$ and calculate the correlation r^*^ between $Y_{i1}^{*}$ and $Y_{i2}^{*}$.
3. Regress $Y_{i2}^{*}$ on $Y_{i1}^{*}$ and keep the regression coefficients $\hat{\beta }$ and the residuals ${\hat{ɛ}}_{i}$. Note that since ${\mathrm{sd}}_{1}^{*}={\mathrm{sd}}_{2}^{*}=1$, it follows that ${\hat{\beta }}_{i}=r^{*}$ and ${\hat{ɛ}}_{i}=Y_{i2}^{*}-r^{*}Y_{i1}^{*}$. Also note that the residuals are uncorrelated to $Y_{i1}^{*}$ and have variance 1 − r^*2^.
4. Generate $Y_{i3}^{*}=Y_{i1}^{*}r+{\hat{ɛ}}_{i}\sqrt{1-r^{2}}{\left[\sqrt{1-r^{*2}}\right]}^{-1}$. Note that $\mathrm{var}\left(Y_{i3}^{*}\right)=1$ and its correlation with $Y_{i1}^{*}$ is *r*.
5. Generate the pseudo baseline as follows: $Y_{\mathrm{Bi}}=Y_{i1}^{*}{\mathrm{sd}}_{B}+{\bar{Y}}_{B}$.

One can immediately verify that the pseudo baseline measurements have mean ${\bar{Y}}_{B}$ and standard deviation *sd*_*B*_.

1. Generate the pseudo follow‐up outcome as follows: $Y_{\mathrm{Fi}}=Y_{i3}^{*}{\mathrm{sd}}_{F}+{\bar{Y}}_{F}$.

Similarly, the pseudo follow‐up outcomes have mean ${\bar{Y}}_{F}$ and standard deviation *sd*_*F*_ and *cor*(*Y*_*Bi*_, *Y*_*Fi*_) = *r*.

This algorithm can be easily carried out in standard statistical software. In the Supporting Information we show how this algorithm can be carried out in R^31^ and SAS.^32^ The pseudo IPD can now be analysed using the LMM methods for IPD of Sections 3 and 4.

In practice, the group correlations are rarely reported. However, the mean change from baseline, with the standard deviation or standard error are more often provided. When the standard deviation at baseline, at follow‐up and the change from baseline *sd*_*Change*_ are reported, the group correlation can be directly calculated as follows:

(6) $$r=\frac{{\mathrm{sd}}_{B}^{2}+{\mathrm{sd}}_{F}^{2}-{\mathrm{sd}}_{\text{Changescores}}^{2}}{2{\mathrm{sd}}_{B}{\mathrm{sd}}_{F}}$$

For more details see the Cochrane Handbook,^33^ Chapter 16. Alternatively, if the standard error of the difference between groups in mean change scores is provided and the pre/post correlations are assumed to be equal between the two groups; the correlation can be calculated as:

(7) $$r=\frac{{\mathrm{sd}}_{\mathrm{BT}}^{2}/n_{T}+{\mathrm{sd}}_{\text{FT}}^{2}/n_{T}+{\mathrm{sd}}_{\mathrm{BC}}^{2}/n_{C}+{\mathrm{sd}}_{\mathrm{FC}}^{2}/n_{C}-{\mathrm{se}}_{\text{difChangescores}}^{2}}{2{\mathrm{sd}}_{\mathrm{BT}}{\mathrm{sd}}_{\text{FT}}/n_{T}+2{\mathrm{sd}}_{\mathrm{BC}}{\mathrm{sd}}_{\mathrm{FC}}/n_{C}}$$

where *T* and *C* are the indexes for treatment and control group, respectively.^21^ When the group correlation cannot be derived from the available data, one could resort to imputation methods.^34^, ^35^, ^36^

## 6 APPLICATION OF THE METHODS TO THE DATA

We generated pseudo IPD baselines and outcomes for the aggregate hypertension data of Table 1, the aggregate hypertension dataset with artificial baseline imbalance and the AD of the obstructive sleep apnea example (given in the Supporting Information). Using these pseudo IPD we subsequently fitted the LMM models (1), (2), (3), (4)) discussed in Section 3; stratified study models and random study models, both with and without the interaction between treatment and baseline measurements. For the stratified models including the interaction term of baseline with the treatment effect, we assumed an unstructured variance‐covariance matrix for the two random effects. For the random study models, we centered the groups when specifying the random effects, and assumed independent random effects due to memory issues. The parameters in the models were estimated using restricted maximum likelihood (REML^37^).

We fitted all models using the LMM program of SAS, PROC MIXED because SAS has explicit options for modeling the within‐study residual variances and allows for additional flexibility using different methods to calculate the degrees of freedom and hence confidence intervals of the treatment effect. We used two different approaches, the default method where the degrees of freedom are calculated using the “between within” method in SAS, as it was the method also used in our previous work and also the Satterthwaite approximation method,^38^ following the recommendations of Legha et al,^11^ who performed an extensive simulation study comparing the models in Section 3 under different CI derivations options.

In the Supporting Information we provide details on the SAS code and on how to fit the same models in R using nlme.^39^ For comparison purposes with the results of Riley et al,^21^ we present only the CIs derived using the between‐within method.

### 6.1 Results of the hypertension example with baseline balance

Results of the analyses using the pseudo IPD generated from the aggregate data on hypertension were compared with the two‐stage IPD meta‐analysis results of Riley et al,^21^ who (unlike us) had access to the original IPD. As mentioned a two‐stage IPD meta‐analysis is very similar to the stratified study model of Equation (1) assuming equal residual variances between the treatment and the control group per study, that is, study‐specific variances: $\sigma_{\mathrm{ik}}^{2}=\sigma_{i.}^{2}$. We also performed a two‐stage ANCOVA using the pseudo IPD. For completeness we also present the results of an AD meta‐analysis using the change scores.

The results for the baseline balanced example are shown in the top two rows of Table 2. Across all competing models, the treatment effect estimates were negative indicating that the hypertension treatment reduced systolic blood pressure values.

**TABLE 2:** Meta‐analysis results of *summary treatment effect* using the pseudo IPD approach compared with the true IPD and standard AD modeling approaches of Riley et al^21^

			Pseudo IPD meta‐analysis				True IPD meta‐analysis	AD meta‐analysis
			One‐stage ANCOVA				ANCOVA model results as described in Riley et al^21^	Change scores
Dataset	Model	Results	$\sigma_{\mathrm{ik}}^{2}$	$\sigma_{i.}^{2}$	$\sigma_{.k}^{2}$	*σ*^2^	$\sigma_{i.}^{2}$	
Hypertension (balanced)	Stratified study (Equation 1)	${\hat{\beta }}_{1}$	−10.17	−10.17	−10.34	−10.34	−10.17	−10.10
		SE	0.93	0.93	0.98	0.98	0.93	0.99
		95% CI	(−11.99, −8.34)	(−12.27, −8.06)	(−12.26, −8.43)	(−12.26, −8.43)	(−12.28, −8.05)	(−12.33, −7.87)
		$\tau_{1}^{2}$	7.12	7.11	8.17	8.17	7.15	6.56
	Random study (Equation 2)	${\hat{\beta }}_{1}$	−10.45	−10.46	10.56	−10.56		
		SE	0.99	0.99	1.01	1.01		
		95% CI	(−12.39, −8.52)	(−12.39, −8.53)	(−12.53, −8.58)	(−12.54, −8.59)		
		$\tau_{1}^{2}$	8.61	8.62	9.13	9.16		
Hypertension (imbalanced)	Stratified study (Equation 1)	${\hat{\beta }}_{1}$	−14.57	−14.55	−14.58	−14.57	−14.55	−10.10
		SE	1.65	1.66	1.64	1.65	1.66	0.99
		95% CI	(−17.81, −11.33)	(−18.31, −10.80)	(−17.80, −11.36)	(−17.78, −11.36)	(−18.30, −10.80)	(−12.33, −7.87)
		$\tau_{1}^{2}$	25.28	25.47	25.30	25.19	25.43	6.56
	Random study (Equation 2)	${\hat{\beta }}_{1}$	−14.45	−14.46	−14.49	−14.48		
		SE	1.65	1.65	1.63	1.63		
		95% CI	(−17.69, −11.20)	(−17.67, −11.20)	(−17.69, −11.29)	(−17.68, −11.29)		
		$\tau_{1}^{2}$	25.34	25.23	25.10	24.99		

Abbreviations: CI, confidence interval; SE, standard error; $\sigma_{\mathrm{ik}}^{2}$, study‐ and arm‐specific variances; $\sigma_{i.}^{2}$, study‐specific variances; $\sigma_{.k}^{2}$, two variance parameters, one for control and one for treatment; *σ*^2^, one overall variance.

The estimated treatment effect and corresponding standard error of the one‐stage pseudo IPD ANCOVA analysis assuming study‐specific residual variances, were identical to the results based on the analysis of the true IPD by Riley et al^21^; −10.17 (SE = 0.93) vs −10.17 (SE = 0.93). There are slight differences in the 95% CIs as they were derived by different methods; under the Satterthwaite correction method were slightly wider. In addition, a two‐stage analysis on the pseudo IPD assuming study‐specific residual variances yielded identical results to model (1) and the analysis of the true IPD^21^: a summary treatment effect of −10.17, SE = 0.93.

We compared the AIC values^40^ of different within‐study residual variance structures for the stratified study models and for the random study models. In both model blocks the lowest value was found for the assuming all within‐study residual variances to be free (arm‐specific and study specific; 243 387.2), although AIC values were found to be very similar across the different within‐study variance options, suggesting that one could potentially adopt a simpler model when opting for a more parsimonious model. The study stratified model assuming within‐study variances to be study‐specific had the second lowest AIC value (243 411.9) in that model block and was adopted as the final model. This model showed a summary treatment effect of −10.17 [95% CI: (−12.27, −8.06)], indicating that on average antihypertension treatments have a positive effect on SBP levels, reducing them by 10.17 mmHg more compared with control/no treatment.

The last column of Table 2 shows the results of the standard AD analysis following a change scores approach; a summary treatment effect −10.10 [95% CI: (−12.33, −7.87)], slightly lower than the ANCOVA estimate using one‐stage or two‐stage pseudo IPD.

### 6.2 Results of the hypertension example with baseline imbalance

For the aggregate data with baseline imbalance, the effect of the active hypertension treatments compared with control is more pronounced (bottom rows of Table 2). We adopt the stratified study model as the final model which produces a summary treatment effect of −14.55 [95% CI: (−18.31, −10.80)], identical to the ANCOVA result of the true IPD presented in Riley et al.^21^ Using a two‐stage analysis of the pseudo IPD assuming study‐specific residual variances resulted also in a summary treatment effect of −14.55 [95% CI: (−18.30, −10.80)].

The results of the pseudo IPD analysis were substantially different from the standard AD meta‐analysis of change scores, because of the induced baseline imbalance.

### 6.3 Including the interaction between baseline and treatment effect

To investigate potential treatment‐by‐baseline modification, we included the interaction term *β*_3_ between baseline and treatment effect in the pseudo IPD LMM models. We compared the pseudo IPD models (3) and (4) with the two‐stage IPD meta‐analysis of Riley et al^21^ with interaction, and with a random‐effects meta‐regression of the final values on the mean baseline of the treatment group. The estimate obtained from the AD meta‐regression is actually comparable to the *β*_4_ term, which quantifies the across‐trial interaction. In the results we focus on the within‐trial interaction estimate *β*_3_ which reflects the treatment‐by‐baseline interaction.

In the balanced example case, the derived pseudo IPD ANCOVA interaction term under the stratified study model assuming all within‐study residual variances to be free was equal to −0.09 [95% CI: (−0.17, −0.01)], providing some evidence that the treatment effect is slightly higher for the more severe hypertensive patients at baseline with higher SBP baseline values (top row of Table 3). In addition, the result from model (3) assuming study‐specific residual variances was found to be identical to the two‐stage model fitted in Riley et al,^21^ −0.09 (SE: 0.038). Using a two‐stage analysis of the pseudo IPD assuming study‐specific residual variances in SAS yielded a summary treatment‐by‐baseline interaction effect of −0.09 [95% CI: (−0.18, −0.00)]. We also replicated the two‐stage analysis in STATA using the DerSimonian‐Laird method^41^ to combine the effects, where the results were found identical to the analysis in Riley et al.^21^

**TABLE 3:** Meta‐analysis results of *interaction* of baseline with treatment using the pseudo IPD approach compared with the true IPD and standard AD modeling approaches of Riley et al^21^

			Pseudo IPD meta‐analysis				True IPD meta‐analysis	AD meta‐analysis
			One‐stage ANCOVA: including the interaction between baseline and treatment				ANCOVA model results as described in Riley et al^21^	Meta‐regression
Dataset	Model	Results	$\sigma_{\mathrm{ik}}^{2}$	$\sigma_{i.}^{2}$	$\sigma_{.k}^{2}$	*σ*^2^	$\sigma_{i.}^{2}$	Using ${\bar{Y}}_{\mathrm{BTi}}$
Hypertension (balanced)	Stratified study (Equation 3)	${\hat{\beta }}_{3}$	−0.09	−0.09	−0.10	−0.10	−0.09	−0.16
		SE	0.04	0.04	0.04	0.04	0.03	0.05
		95% CI	(−0.17, −0.01)	(−0.17, −0.01)	(−0.18, −0.01)	(−0.18, −0.01)	(−0.16, −0.03)	(−0.28, −0.04)
		$\tau_{3}^{2}$	0.01	0.01	0.01	0.01	0.01	3.16
	Random study (Equation 4)	${\hat{\beta }}_{3}$	−0.09	−0.09	−0.10	−0.10		
		SE	0.04	0.04	0.04	0.04		
		95% CI	(−0.17, −0.01)	(−0.17, −0.01)	(−0.18, −0.01)	(−0.17, −0.02)		
		$\tau_{3}^{2}$	0.01	0.01	0.01	0.01		
Hypertension (imbalanced)	Stratified study (Equation 3)	${\hat{\beta }}_{3}$	−0.09	−0.09	−0.10	−0.10	−0.09	0.20
		SE	0.04	0.04	0.04	0.04	0.03	0.11
		95% CI	(−0.17, −0.01)	(−0.17, −0.01)	(−0.18, −0.01)	(−0.18, −0.01)	(−0.16, −0.03)	(−0.76, 0.50)
		$\tau_{3}^{2}$	0.01	0.01	0.01	0.01	0.01	47.85
	Random study (Equation 4)	${\hat{\beta }}_{3}$	−0.09	−0.09	−0.10	−0.11		
		SE	0.04	0.04	0.04	0.0		
		95% CI	(−0.17, −0.01)	(−0.17, −0.01)	(−0.18, −0.01)	(−0.19, −0.02)		
		$\tau_{3}^{2}$	0.010	0.011	0.012	0.012		

Abbreviations: CI, confidence interval; SE, standard error; ${\bar{Y}}_{\mathrm{BTi}}$, mean baseline SBP value of the treated group per trial; $\sigma_{\mathrm{ik}}^{2}$, study‐ and arm‐specific variances; $\sigma_{i.}^{2}$, study‐specific variances; $\sigma_{.k}^{2}$, two variance parameters, one for control and one for treatment; *σ*^2^, one overall variance.

The meta‐regression results using the mean baseline value of the treatment group were higher compared with the pseudo IPD ANCOVA model (−0.16 vs −0.09).

The estimates of the interaction effect in the imbalanced baseline dataset using the pseudo IPD were found to be very similar to the ones in the balanced case. However, the meta‐regression estimate was in the opposite direction of the effect compared with the ANCOVA pseudo IPD results. The across‐trial interaction as estimated from a standard AD meta‐analysis can differ from the within‐trial interaction, that is, the difference in treatment effect of two patients in the same study differing one unit at baseline, as estimated from a true IPD or pseudo IPD meta‐analysis. The assumption that they are the same is often not plausible due to the fact that across‐trial interaction can suffer from confounding.^5^ This phenomenon is called ecological or aggregation bias. Therefore the across‐trials interaction should be carefully interpreted. Also note that the statistical power for the estimation of the within‐trial interaction is usually much larger than for the across‐trials interaction, as reflected by the standard errors (Table 3).

### 6.4 Results of the obstructive sleep apnea example

In this second example, it was possible to calculate the group correlations (assumed to be equal between active and sham) using Equation (7); the derived correlations values varied slightly across studies [median: 0.498, IQR: 0.496‐0.503]. We additionally performed sensitivity analyses by imputing three values of *r* (0.5, 0.6 and 0.7), to simulate cases where deriving the correlations from available data would not be possible. The R package ggplot2^42^ was used to visualize the results of the competing models.

**FIGURE 2:**
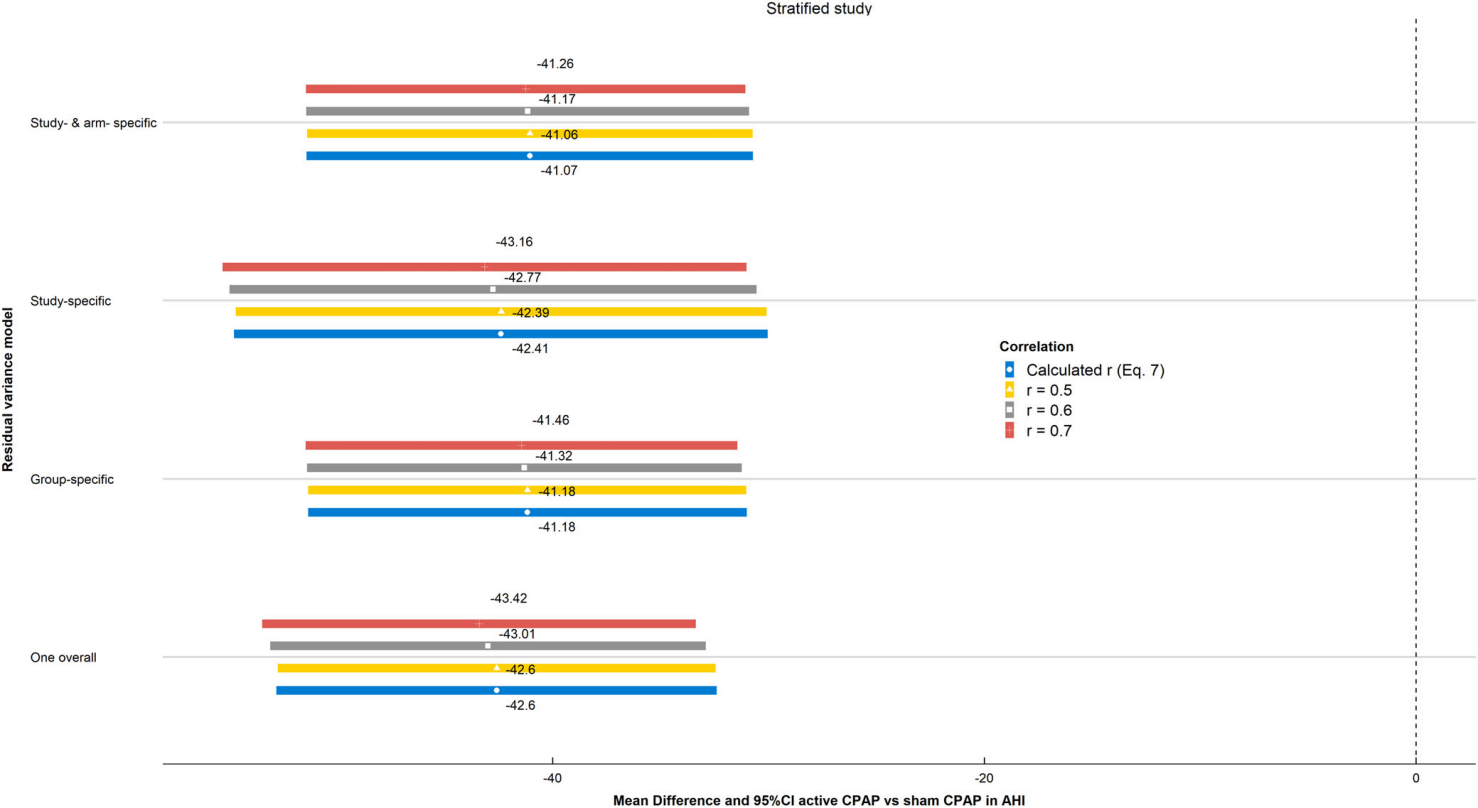
Obstructive sleep apnea meta‐analysis results: estimates of overall mean difference of active CPAP vs sham and 95% CI in AHI across different residual variance models and varying group correlation coefficients between baseline and follow‐up values [Colour figure can be viewed at wileyonlinelibrary.com]

Figure 2 shows the results of the one‐stage stratified study model assuming different options for the within‐study residual variances. Results consistently showed that CPAP statistically significantly reduces AHI compared with the sham device (∼41 events/hour). When *r* was calculated from the summary data (blue line/circle estimate), the point estimates across competing models varied slightly between 41 and 42 less events per hour in favor of active CPAP. The lowest AIC value was found for the most flexible model assuming arm and study residual variances to be free (AIC = 2273). Overall, AIC values did not differ greatly across the models hence simpler structures can also be adopted, for example, study‐specific within‐study residual variances model.

The point estimates and 95% CIs were found to vary little across the imputed values of *r*, and the differences were not deemed to be clinically significant. The differences within the blocks of the more flexible modeling options (study‐ and arm‐specific, and study‐specific within‐study residual variables) were more pronounced compared with the results of the more restricted models (group specific and one overall variance). Overall, the results based on the different imputed values within the same model block and across models did not seem to materially differ.

For this example, no direct comparison is feasible with the true IPD, thus we present the results of the one‐ and two‐stage pseudo IPD analysis (using the calculated *r* value) and the original meta‐analysis,^22^ and compare them with each other (Table 4). The one‐stage stratified study model and the two‐stage ANCOVA model, which form a natural comparison with one another, produced identical results when rounded in two decimal places (rows 3‐4, Table 4). The point estimate of the standard AD change score analysis was larger compared with the ANCOVA results of the pseudo IPD, which may be explained by the negative correlation of the change scores with the baseline scores and the worse baseline of the subjects randomized in the active group. Generating the pseudo IPD enabled us to explore the interaction of baseline values with the treatment effect which in this example was found to be statistically significant (last two rows of Table 4), suggesting that the treatment effect is higher for the patients randomized in the active CPAP arm who were found to suffer more at baseline compared to the control patients.

**TABLE 4:** Meta‐analysis results of *summary treatment effect and interaction effect* using the pseudo IPD approach compared with standard change score AD methods

Approach	Method	Estimate	Standard Error	95% CI
Standard AD	Difference in change scores as in Balk et al^22^	−46.39	5.39	(−56.97, −35.81)
Pseudo IPD	One‐stage ANCOVA^a^ Equation (1)	−42.41	5.23	(−54.77, −30.05)
	Two‐stage ANCOVA Equation (5)	−42.41	5.23	(−54.77, −30.04)
	One‐stage ANCOVA interaction effect Equation (3)	−0.40	0.07	(−0.54, −0.25)
	Two‐stage ANCOVA interaction effect	−0.40	0.07	(−0.54, −0.25)

Abbreviations: CI, confidence interval, Assumed *r*_*CPAP*_ = *r*_*sham*_; ${\bar{Y}}_{\mathrm{BTi}}$, mean baseline AHI values of the treated group per trial.

^a^ A study‐stratified model with study specific variances was used.

## 7 DISCUSSION

We have shown how aggregate data from comparative studies of continuous outcomes measured at baseline and follow‐up can be analysed by generating pseudo IPD. These pseudo IPD enable us to use the complete palette of techniques available for IPD meta analyses. In particular, we are able to (a) perform an ANCOVA, where we can adjust for baseline imbalances between treatment and control groups and to (b) explore interactions between baseline values and treatment effects. Different modeling approaches of increasing complexity can be applied by using the linear mixed model (LMM) framework. Since the LMM analyses are likelihood‐based, one‐stage and two‐stage results derived using the pseudo IPD baseline and follow‐up outcomes are identical to the ones of the original IPD. The proposed methods can be applied in any standard statistical software therefore eliminating the need for training on a special purpose meta‐analytic software.

In this article we have described modeling situations of comparing two treatment groups using the follow‐up and baselines values. However, the LMM is a broad framework which offers rather staightfoward extensions of this work; the algorithm is directly generalizable to repeated measures meta‐analysis and to multiple‐treatments meta‐analysis. Extension of the method for meta‐analysis of cross‐over trials is also applicable with some modifications albeit beyond the scope of this work. In addition, incorporation of non‐linear covariates or non‐linear interactions of treatment with continuous covariates could be a topic of future research as in this work we included the baseline (our covariate of interest) as a linear term in the ANCOVA model. Our algorithm could be extended to incorporate other covariates than only the baseline if the required summary statistics are available, in this case the variance‐covariance matrix per group. These summaries are practically never reported however it is much easier to request them from the authors compared to the true IPD, as no privacy issues are involved. Bonofiglio and authors recently proposed a similar approach under distributed computing setting framework using only IPD summaries to recreate the marginal distributions of the original IPD considering eight baseline predictors in a multivariable logistic regression model.^43^

The proposed approach successfully addresses the problem of IPD disclosure which is seldom possible due to various reasons with respect to data privacy and data security. In the case of continuous outcomes measured at baseline and follow‐up often the sufficient aggregate data may be only partially available; for example often only means and standard deviations at baseline and mean change from baseline scores with the respective standard deviation or standard error are reported. Less frequently the mean and the standard deviation values at follow‐up are provided. In that case, we could resort to algebraic calculations or imputation methods.^34^, ^36^ In principle, the minimally required set of aggregate data is the means and standard deviations at baseline and follow‐up and also the standard deviation of the change from baseline. If these three standard deviations are provided, the correlation coefficient of baseline and follow‐up can be calculated.^33^ If one of these standard deviations are missing, they can potentially be algebraically extracted by other commonly reported summary statistics, for example, confidence interval of mean difference, standard error of mean difference, paired *t* test or a *p*‐value from a paired *t*‐test.^44^, ^45^, ^46^ In cases where the post‐baseline standard deviation is missing, it is common practice to assume it equal to the standard deviation at baseline and thus enable the calculation of the within‐group correlation. Another commonly used approach is to impute the missing SDs at post‐baseline from other similar studies, with respect to study and patient characteristics, included in the meta‐analysis. Recently, Weir and colleagues^36^ proposed 15 methods for addressing missing standard deviations (and by extension group correlations) in continuous data meta‐analysis, building on the empirical review of Wiebe and colleagues in 2006.^34^ Interested readers are referred to these reviews as a lengthy description of available methods for calculating or imputing the missing summary data is beyond the scope of this work. We also encourage contacting the authors of the original studies to provide the aggregate data also at follow‐up, when confidentiality issues prohibit the direct provision of IPD.

We compared our pseudo IPD approach to standard meta‐analytic approaches for aggregate data: random effects meta‐analysis using change scores and meta‐regression of the final scores on the baseline values of the treatment group to compare their performance with the pseudo IPD models. In case of imbalanced baseline values, the AD methods based on change scores tend to provide biased treatment and interaction effect estimates compared with the pseudo IPD ANCOVA methods.

Another advantage of the pseudo IPD approach is that it allows us to make more realistic and flexible assumptions regarding the within‐study residual variances. In the absence of computational or estimations issues, we propose to use a realistic structure of the within‐study residual variance. This flexibility is not possible in the standard AD analysis. Moreover, the standard AD assumes the standard errors of the treatment effects to be fixed and known, while using pseudo IPD ANCOVA methods may account for the fact that these are estimated.

When the appropriate AD are available (ie, two means, standard deviations and correlation per group), we strongly recommend our proposed methodology to construct the pseudo IPD and perform an ANCOVA, if needed including the treatment‐by‐baseline interaction term. The advantage of our method is highlighted particularly in the case of baseline imbalance and in the case of treatment‐baseline interaction, as the standard AD methods for interaction are known to suffer from low power and the potential of ecological‐bias.

## CONFLICT OF INTEREST

The authors declare no potential conflict of interests.

## Supporting information


**Appendix** **S1.** Supporting Information.

**TABLE S1** Aggregate data of the 8 trials included in the meta‐analysis of Balk et al^22^

## Data Availability

The data that supports the findings of this study are available in the supplementary material of this article.
