# Supplementary material for: Meta‐analysis of continuous outcomes: Using pseudo IPD created from aggregate data to adjust for baseline imbalance and assess treatment‐by‐baseline modification
Source: Res Synth Methods. 2020 Jul 25;11(6):780–94. doi: 10.1002/jrsm.1434 (PMC7754323; doi:10.1002/jrsm.1434)
Supplement: Supplementary file 2 — TABLE S1 Aggregate data of the 8 trials included in the meta‐analysis of Balk et al22 [file JRSM-11-780-s002.pdf]

TABLE S1 Aggregate data of the 8 trials included in the meta-analysis of Balk *et al.*[22]

		Number of subjects		AHI index at baseline		AHI index at follow-up		Reported correlation	Calculated correlation using Eq. (7)
				Treatment	Control	Treatment	Control		Equal between treatment and control groups
ID	Trial name	Treatment	Control	Mean (SD)	Mean (SD)	Mean (SD)	Mean (SD)		
1	Egea 2008	27	29	43.7 (22.9)	35.3 (16.7)	10.8 (11.4)	28.0 (24.8)	-	0.4979
2	Haensel 2007	25	25	65.9 (28.6)	57.5 (32.1)	3.5 (3.4)	53.4 (32.9)	-	0.4981
3	Loredo 1999	23	18	56.4 (24.1)	44.2 (25.3)	3.3 (3.8)	28.3 (22.7)	-	0.4442
4	Mills 2006	17	16	65.0 (34.0)	61.2 (41.0)	2.6 (2.4)	57.3 (41.0)	-	0.4969
5	Loredo 2006	22	19	65.9 (28.6)	57.5 (32.1)	3.0 (4.7)	52.5 (37.5)	-	0.5704
6	Norman 2006	18	15	66.1 (29.1)	53.9 (29.8)	3.4 (3.0)	50.1 (32.1)	-	0.4967
7	Becker 2003	16	16	62.5 (17.8)	65.0 (26.7)	3.4 (3.1)	33.4 (29.2)	-	0.5025
8	Spicuzza 2006	15	10	55.3 (11.9)	59.2 (17.3)	2.1 (0.3)	57.0 (8.6)	-	0.5052

AHI: Apnea-hypopnea index, SD: standard deviation

For Review Only
